# Timescale effects on the environmental control of carbon and water fluxes of an apple orchard

**DOI:** 10.1002/ece3.3633

**Published:** 2017-11-29

**Authors:** Leonardo Montagnani, Damiano Zanotelli, Massimo Tagliavini, Enrico Tomelleri

**Affiliations:** ^1^ Faculty of Science and Technology Free University of Bolzano Bolzano Italy; ^2^ Forest Services Autonomous Province of Bolzano Bolzano Italy; ^3^ The European Academy of Bozen/Bolzano (EURAC) Bolzano Italy

**Keywords:** environmental constraints, evapotranspiration, hysteresis, modeling, net ecosystem exchange, phase, timescales

## Abstract

Model parameterization and validation of earth–atmosphere interactions are generally performed using a single timescale (e.g., nearly instantaneous, daily, and annual), although both delayed responses and hysteretic effects have been widely recognized. The lack of consideration of these effects hampers our capability to represent them in empirical‐ or process‐based models. Here we explore, using an apple orchard ecosystem in the North of Italy as a simplified case study, how the considered timescale impacts the relative importance of the single environmental variables in explaining observed net ecosystem exchange (NEE) and evapotranspiration (ET). Using 6 years of eddy covariance and meteorological information as input data, we found a decay of the relative importance of the modeling capability of photosynthetically active radiation in explaining both NEE and ET moving from half‐hourly to seasonal timescale and an increase in the relative importance of air temperature (*T*) and VPD. Satellite NDVI, used as proxy of leaf development, added little improvement to overall modeling capability. Increasing the timescale, the number of variables needed for parameterization decreased (from 5 to 1), while the proportion of variance explained by the model increased (*r* ^2^ from 0.56–0.78 to 0.85–0.90 for NEE and ET respectively). The wavelet coherence and the phase analyses showed that the two variables that increased their relative importance when the scale increased (*T*, VPD) were not in phase at the correlation peak of both ET and NEE. This phase shift in the time domain corresponds to a hysteretic response in the meteorological variables domain. This work confirms that the model parameterization should be performed using parameters calculated at the appropriate scale. It suggests that in managed ecosystems, where the interannual variability is minimized by the agronomic practices, the use of timescales large enough to include hysteretic and delayed responses reduces the number of required input variables and improves their explanatory capacity.

## 1 INTRODUCTION

Tree crops represent a significant component of the global carbon cycle. They cover more than 10 million hectares in Europe (FAOSTAT, 2010) and are the most relevant crop class in southern Europe after the cereals. Their role in the carbon balance is questioned: They are a strong sink during the vegetation season, but much of the organic material which they assimilate is shortly released (Nemeth, Lambrinos, & Strik, 2017; Zanotelli, Montagnani, Manca, Scandellari, & Tagliavini, 2015).

Their interaction with the atmosphere takes place at multiple timescales: trees sequester carbon through photosynthesis and release it through different pathways: (1) autotrophic and (2) heterotrophic respiration, (3) export from the field and storage in different forms and places (as oil, vine, fruit and fruit juices, and timber in some cases), with different patterns and places of release. In addition, they sequester carbon in perennial vegetative organs, with a turnover time of decades, or even centuries as in the case of olive trees. All these processes do not occur at the same rate across the year (Zanotelli, Montagnani, Manca, & Tagliavini, 2013).

The environmental control on assimilation is expected to take place at different timescales: Solar radiation, air temperature, and soil water content (SWC) can influence plant physiology almost instantaneously, but they can influence plant behavior also through slower processes, such as stomatal opening, chlorophyll formation and depletion, and leaf and fruit development, which operate at temporal scales ranging from few minutes to a whole season.

In addition, a number of possibly overlapping effects take place at different timescales: Namely, the stomatal response to soil drought can be combined with the water pressure deficit for determining transpiration (Barlett, Klein, Jansen, Choat, & Sack, 2016; Novick et al., 2016; Tuzet, Perrier, & Leuning, 2003) the variation in leaf area can be combined with that of chlorophyll content and the amount of fruits for the definition of the maximal photosynthetic capacity (Giuliani, Nerozzi, Magnanini, & Corelli, 1997; Navarro et al., 2008; Tartachnyk & Blanke, 2004; Zanotelli, Scandellari, Bastos de Melo, & Tagliavini, 2016). All these potentially overlapping processes differentially influence the biological fluxes. As a general consequence, the presence of almost immediate and delayed responses, and of overlapping phenomena, gives rise to a number of entangled and hysteretic responses (Niu et al., 2011; Wohlfahrt & Galvagno, 2017; Zhang, Manzoni, Katul, Porporato, & Yang, 2014) hampering our modeling capability.

In fact, a common problem in ecophysiological studies is the high degree of overlapping between the temporal patterns of environmental variables. Radiation, temperature, and vapor pressure deficit show a high degree of self‐correlation, with solar activity being the main driver for these processes. The plant organs perceive these stimuli in different, sometime contrasting ways. Shortwave radiation is the resource determining the photosynthetic process and favoring the opening of stomatal guard cells. The vapor pressure deficit has an opposite effect in controlling stomatal opening, although it is difficult to disentangle its effects from those of temperature, the driving parameter for its computation (Duursma et al., 2014).

The linkage between plant gas exchange and environmental constraints, particularly temperature and soil water availability, is a well‐established concept in plant physiological ecology (Schwarz et al., 2004; Yi et al., 2010). Radiation and vapor pressure deficit are frequently used as further limiting factors in several biogeochemical models, particularly in those based on light use efficiency, like the Prelued model (Mäkelä et al., 2008) and in remote sensing based models, like MOD 17 (Running, Thornton, Nemani, & Glassy, 2000). However, it is still to be defined the extent of the direct limitation of the environmental processes, or the mediated effect through vegetation modifications in a slower response. For instance, Michaletz, Cheng, Kerkhoff, and Enquist (2014) have suggested that the variability of gross primary productivity is driven by a broad range of biotic and abiotic factors, mainly through changes in vegetation phenology and physiological processes, and not through direct environmental limitations.

Modeling growth and evapotranspiration have become an important challenge in agriculture, as it might predict future scenarios of crop productivity and resource availability under changing climatic conditions. Current knowledge of interactions between environmental drivers and fruit tree physiology mainly derives from cuvette assessment of gas exchange at leaf level (e.g., Giuliani et al., 1997). This approach gives detailed information on short‐term effects of environmental forcing, but, as measurements are generally carried out for short periods, it requires an upscaling for medium‐ and long‐term (weeks to years) assessments (Jarvis & McNaughton, 1986). On the contrary, the eddy covariance (EC) technique allows better insights into physiological responses at different timescales, although it does not achieve the same level of detail as cuvette‐based studies do.

The necessity to use data for model parameterization at the appropriate scale is a concept widely acknowledged in the modeling community. To date, only a few studies addressed the question about the modeling parameter variation at the different timescales. Analyzing the interactions in temperate and tropical ecosystems, these studies consistently found that environmental variation is responsible for short‐term variation in CO_2_ exchange but biological variability is responsible for longer‐term variation in CO_2_ exchange (Braswell, Sacks, Linder, & Schimel, 2005; Keenan, Davidson, Moffat, Munger, & Richardson, 2012; Richardson, Hollinger, Aber, Ollinger, & Braswell, 2007; Wu et al., 2015).

Do the same holds true for crops? In managed ecosystems and in fruit orchards in particular, the interannual variability in productivity is, in contrast, maintained low by management practices (Ceschia et al., 2010; Scandellari et al., 2016; Zanotelli et al., 2015). Although several factors can affect the amount of fruits that trees bear every year, different winter pruning intensities across the years allow setting a relative homogeneous number of flower buds, with the aim of minimizing the interannual variation in fruit yields. Other management practices, including the regular application of water and nutrients through irrigation and fertilization, make the apple orchard an ideal, simplified ecosystem for the study of interactions at subannual timescales.

Therefore, we conceived this study under the general hypothesis that increasing the timescale, the correlation between environmental drivers and fluxes change and possibly increases. In fact, extending the integration time, both hysteretic and delayed responses are captured, but long‐term biological changes are not expected as a result of agronomic practices.

In this study, thanks to the availability of a 6‐year dataset of gas exchange measurements obtained through the EC technique in an apple orchard, we analyzed the evapotranspiration (ET) and the net ecosystem exchange (NEE) responses to environmental drivers at different timescales, from half‐hourly to multiannual, and addressed the two following questions:

1. Does the relation between environmental drivers and biological fluxes change across the timescales?
2. Can we improve the modeling capability of fluxes showing a hysteretic response to environmental drivers by increasing the time of integration?

Addressing these questions will improve our understanding of the interaction between climate forcing and the productivity of a commercially relevant crop type in the context of climate change, and inductively will give indications for the wider ecosystem modeling perspective. To answer these questions, we firstly analyzed the temporal patterns of the different variables. Then, by the use of the wavelet coherence analysis and the analysis of phases, we analyzed the strength of the correlations and the phases at the correlation peaks. To get insight into the shape of the specific relationships, we developed new empirical equations to describe the correlations at the different scales and we obtained the correlation strength at the different time steps. Finally, we obtained synthetic equations making use of a minimum of variables to describe the observed fluxes.

## 2 MATERIAL AND METHODS

### 2.1 Study site

Measurements were taken from 2009 to 2014 in an experimental site located in the municipality of Caldaro (46°21′N, 11°16′E, 240 m a.s.l.), located at the bottom of the Adige Valley (North Italy), within a commercial apple growing district. Apple (*Malus domestica* Borkh.) trees of the Fuji cultivar grafted on dwarfing rootstocks were planted in 1999 in rows at a distance of 1 m along the row and 3 m between rows. Orchard management is carried out according to organic farming guidelines.

In the past, the site was formerly a bog, periodically inundated and after land reclamation (around 1826), the area was kept as a pasture with mulberry trees until the spread of apple cultivation in the sixties of the last century. The water table, controlled by an artificial drainage system, ranges for most of the time between 1.20 and 1.85 m deep. Due to capillary rise, moisture is therefore relatively high, at least in the lower part of the soil layer where roots develop. In addition to precipitation, in late winter, to prevent frost damages, and occasionally in summer, the site is irrigated by overhead sprinklers.

The soil (average of the upper 0–60 cm layer) consists of 11% sand, 44.5% clay and silt. Soil pH in pore water ranges from 7.2 (in the upper 0–20 cm) to 7.6 (between 40 and 60 cm soil depth). Details on carbon allocation and net ecosystem carbon balance can be found in Zanotelli et al. (2013) and in Zanotelli et al. (2015).

### 2.2 Instrumental set‐up

Eddy covariance and meteorological instrumentation were installed in spring 2009. The data period spanning from 2009 to 2014 was used in this study. EC instrumentation consists of a sonic anemometer Gill R3, Limington, UK, and a Li 7000 CO_2_/H_2_O infrared gas analyzer (Li7000, LiCor Biosciences, US, LiCor henceforth), kept in a temperature controlled box. The tower is 8 m high, and air intake occurs through a 12 m polyethylene tube, 4 mm inner diameter, and one Acro 50 Pall filter, replaced each second week. Zero reference was given by chemicals (Ascarite 2 and magnesium perchlorate). At the beginning of 2013, the analyzer was replaced by a Li7200 (LiCor). The new analyzer was horizontally mounted without filtering at 1.5 m distance from the air intake tube.

Meteorological measurements included a CNR1 (Kipp and Zonen, Holland) net radiometer, for short‐ and long‐wave incoming and outgoing radiation; a SKP 215 PAR quantum sensor (Skye Instruments Ltd., UK); an air temperature and humidity probe HMP110 (Vaisala, Finland); an array of six SWC sensors TDR type CS610 (Campbell Scientific. Inc., USA), of which one was inserted vertically up to 30 cm below ground, and was taken as reference, and the others were used to assess the horizontal and vertical spatial variability. Meteorological data were sampled at 0.1 Hz frequency and collected at 30 min intervals by a CR3000 data logger (Campbell Scientific, USA).

### 2.3 Computational methods

#### 2.3.1 Eddy covariance data analysis

Turbulent flux measurements with the EC technique were performed using EDDYSOFT software (Kolle & Rebmann, 2007; Mauder et al., 2008). The following operative details were applied as follows: (1) No detrending, no high‐ or low‐pass filtering corrections were used; (2) a two‐axis rotation of coordinates was applied each 30 min; (3) the inductance due to the presence of the air intake tube was calculated. The software automatically calculated the lag time for CO_2_ each half hour to maximize the covariances between fluctuations in vertical wind velocity and gas dry mole density.

In addition, the analysis of stationary conditions for CO_2_ turbulent flux and of integral turbulent characteristic following Foken and Wichura (1996) was performed. As a result, half hours for which theoretical concerns existed on Reynolds decomposition because of lack of stationarity or for which the turbulence was not well‐developed and not suitable for further detailed analyses (Göckede et al., 2008) were flagged for their recognition. After quality check of the measurements, flux values collected during nonstationary periods or during periods of not well‐developed turbulence, particularly frequent in winter and at night were excluded. After the removal of low‐quality flagged data, gap filling was calculated according to Reichstein et al. (2005). The online processing tool, hosted at the Max Planck Institute for Biogeochemistry, was used (http://www.bgc-jena.mpg.de/bgc-mdi/html/eddyproc/).

#### 2.3.2 Computation of canopy conductance

To disentangle radiation, temperature and water pressure deficit interactions in their daily patterns, we derived the canopy conductance by inverting the Penman–Monteith equation, and by constraining it using observed evapotranspiration values. We followed the approach used by Pérez‐Priego, Serrano‐Ortiz, Sanchez‐Cañete, Domingo, and Kowalski (2013), assuming a canopy completely coupled with the atmosphere (Jarvis, 1985; Jarvis & McNaughton, 1986) and, hence, evapotranspiration regimes imposed by the atmospheric demand

(1) $$g_{c}=E_{\mathrm{imp}}\left(\frac{\lambda \gamma }{\rho {}_{a}c_{p}}\right)D^{-1}$$

where *g*_c_ is canopy conductance, *E*_imp_ is observed evapotranspiration values, ρ_*a*_ is air density (kg/m^−3^), *c*_*p*_ is the specific heat of air (0.00101 J/K), λ is the latent heat of vaporization of water (2.45 kJ/kg at 293.15 K), γ is the psychrometric constant (0.066 kPa/K), and *D* is the water pressure deficit.

### 2.4 Treatment of data and statistics

#### 2.4.1 Analysis of the relationships of environmental variables and satellite NDVI versus observed ET and NEE fluxes at different timescales

Environmental variables to consider were selected (excepted for diffuse radiation, not available) following Law et al. (2002), Nemani et al. (2003), and Groenendijk et al. (2011), while satellite (MODIS) NDVI was selected as a surrogate of LAI. To assess the effect of environmental variables (PAR, Tair, SWC, VPD) and observed fluxes (ET, NEE) at different timescales, the whole dataset was filled using the online tool as specified above. When the gaps in EC data exceeded 16 days, the whole period was discarded. The seasons winter 2009, winter and spring 2011, and summer 2012 were therefore also excluded from further computation. To fill biweekly NDVI data, the singular spectral analysis (SSA) interpolation procedure was used (Buttlar, Zscheischler, & Mahecha, 2014; Kondrashov & Ghil, 2006; Korobeynikov, 2010; Sifuzzaman, 2009).

Measured half‐hour values were averaged (*T*, VPD, SWC, NDVI) or summed (PPFD) to obtain the value in the selected time interval. Selected representative timescale intervals were the half hour, the day, the month and the season; winter season was considered from December to February, and the other seasons were defined consequently. For visualization purposes, the dataset was divided into subgroups having the same width, and statistics of average, median, 1st and 99th percentile were computed.

#### 2.4.2 Wavelet coherence analysis

The wavelet transform originated in geophysics in the early 1980s for the analysis of seismic signals and is becoming a common tool for analyzing localized variations of power within a time series. By decomposing a time series into time–frequency space, both the dominant modes of variability and how those modes vary in time can be determined.

The wavelet coherence analysis (Cazelles et al., 2008; Sá, Sambatti, & Galvao, 1998; Torrence & Compo, 1998; Torrence & Webster, 1998) is a tool to study the linear relation between two signals by determining the correlation between their spectra. Any analysis based entirely on spectral methods (e.g., the Fourier transform) must ignore any temporal structure of the signal beyond phase information and so coherence cannot give any information on dynamically varying dependence between the signals.

The wavelet decomposition works similar to a spectrum, separating the harmonics in a signal while assigning a “wavelet power” to them, which is proportional to the overall variance. The most important harmonics will be the ones with high wavelet power. To overcome many of the shortcomings of signal analysis based on the Fourier transform, which is caused by essentially neglecting time resolution, the wavelet transform has been established as an important technique in time–frequency analysis in the last two decades.

In recent years, wavelet analysis has been used in many studies of geophysical data, such as river levels, turbulence over plant canopies, and pollutant concentrations (Collineau & Brunet, 1993; Stoy et al., 2013; Terradellas, Soler, Ferreres, & Bravo, 2005; Zeri, Oliveira‐Junior, & Bastos Lyra, 2011) and also to study the interactions between environmental drivers and ecosystem fluxes (Braswell et al., 2005; Wagle et al., 2016). Wavelet coherence analysis is able to synthetically identify the points where two variables are correlated in a two dimensional space representing time and frequency. In this study, we used the “Biwavelet” package (http://biwavelet.r-forge.r-project.org) of the R software (R Development Core Team, 2015), and we selected the Morlet function (Mi et al., 2005). We added the analysis of the phase, following Grinsted, Moore, and Jevrejeva (2004).

#### 2.4.3 Single regression analyses at multiple timescales

To quantify the relations between the environmental variables (VPD, PAR, *T*, and SWC) and the observed biological fluxes (NEE, ET) at different timescales (half hour, day, month, and season), different regression equations were developed and applied in this study. In this analysis, the NEE sign was changed (–NEE) to ease regressions. The regression equations used, tested at all the different timescales considered, are defined below.

The relations of ET and –NEE with PAR (Equations (2) and (3)) were derived from previous works (Montagnani, 2000 for ET and Ruimy, Jarvis, Baldocchi, & Saugier, 1995; for –NEE). The following equations were used as follows:

For ET

(2) $$f=a_{\mathrm{ET}}x\left(1-\text{EXP}\left(-xb_{\mathrm{ET}}\right)\right)$$

For –NEE

(3) $$f=\left(a_{\mathrm{NEE}}xb_{\mathrm{NEE}}\right)/\left(a_{\mathrm{NEE}}x+b_{\mathrm{NEE}}\right)+c_{\mathrm{NEE}}$$

The relations of ET and –NEE with temperature (Equations (4) and (5)) were assumed to be logistic. This assumption is not of general use, as in some ecosystems the relation with the temperature tends to decrease, at least in the case of –NEE, when the temperature increases above a given threshold (see Niu et al., 2011). However, such a decrease was not evident in our dataset, and therefore, the three (for ET) or four parameters (for –NEE, to account for respiration) in the logistic equations for ET and NEE were used.

For ET

(4) $$f=a_{\mathrm{ET}}/\left(1+b_{\mathrm{ET}}\text{EXP}\left(c_{\mathrm{ET}}a_{\mathrm{ET}}x\right)\right)$$

For –NEE

(5) $$f=a_{\mathrm{NEE}}/\left(1+b_{\mathrm{NEE}}\text{EXP}\left(c_{\mathrm{NEE}}a_{\mathrm{NEE}}x\right)\right)+d_{\mathrm{NEE}}$$

The observed relations with the SWC were weak. We therefore used the only suitable regression equation, the linear one, for both ET and –NEE:

(6) $$f=a_{\mathrm{ET}}x+b_{\mathrm{ET}}$$

(7) $$f=a_{\mathrm{NEE}}x+b_{\mathrm{NEE}}$$

Preliminary observations showed that, instead of a decay as it is generally assumed in modeling (e.g., Dolman, 1991), or a decay starting at about 1 kPa (Oren et al., 1999) both NEE and ET have a generally positive relation with VPD, possibly reflecting the ample water availability at the site and the direct evaporation from the soil. It was therefore necessary to develop new equations concerning VPD. These equations are of more general use, as they allow the representation of a decrease, an increase, and a decrease starting from a point of maximum as it is frequently observed (Duursma et al., 2013, 2014; Farquhar, 1978; Franks, Cowan, & Farquhar, 1997). The relation with VPD and ET was as follows

(8) $$f=a_{\mathrm{ET}}x\;\left(1+1/\left(b_{\mathrm{ET}}\text{EXP}\left(xc_{\mathrm{ET}}\right)\right)\right)$$

and with –NEE was as follows

(9) $$f=a_{\mathrm{NEE}}x\;\left(1+1/\left(b_{\mathrm{NEE}}\text{EXP}\left(xc_{\mathrm{NEE}}\right)\right)\right)+d_{\mathrm{NEE}}$$

The same linear equation used to describe the SWC effect on ET and –NEE (Equations (6) and (7)) was also used to assess the correlation between NDVI and observed fluxes.

#### 2.4.4 Multiple regression analysis

In order to test the relative importance of environmental parameters to the carbon and water fluxes, multiple linear regression models with NEE or ET, as the response variables, and PAR, air *T*, VPD soil SWC and NDVI as continuous predictor variables, were built. We used four datasets differing in the temporal scale of both the response and predictor variables: every half‐hour data (*n* = 92,640), daily averages (*n* = 1,930), monthly averages (*n* = 63), and seasonal averages (*n* = 19). The procedure used, starting from the full model (all variables and their interactions), identifies the minimum adequate model by backward deletion procedure (discarding variables with a *p* value > .05). We used the “lm” function from the R statistical computing environment (R Development Core Team, 2015). The relative importance of the significant terms was obtained applying the function “calc.relimp” of the R package “Relaimpo” (Groemping, 2006) using the default options.

## 3 RESULTS

### 3.1 Temporal pattern of the considered variables

#### 3.1.1 Meteorology, soil conditions, NDVI, and observed H_2_O and CO_2_ fluxes

**Figure 1:**
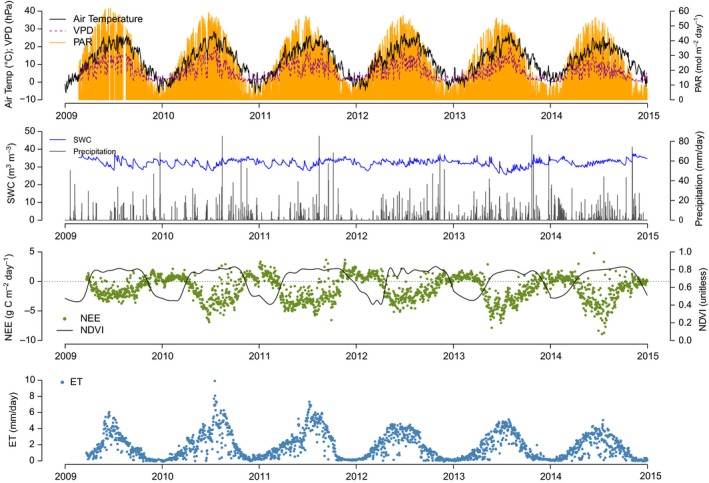
Observed patterns in time along the measurement period (2009–2014) at the daily timescale. Top panel: average air temperature (air Temp), vapor pressure deficit (VPD), and daily cumulative photosynthetic active radiation (PAR); second panel: soil water content and precipitation; third panel: measured NEE and satellite‐derived normalized difference vegetation index (NDVI); bottom panel: evapotranspiration (ET)

Multiannual patterns of observed meteorological and soil variables are shown in Figure 1. During the considered period (January 2009–December 2014), air temperature ranged between −10.6°C and 36.5°C, with an average of 12.3°C. Average annual global radiation was 5,032.5 MJ m^−2^ year^−1^, with a minimum of 4,976.5 MJ m^−2^ year^−1^ during 2014 and a maximum of 5,082.2 during 2012. The lowest annual maximum VPD of (34.3 hPa) was recorded in June 2014, while the absolute maximum was observed in August 2013 (43.7 hPa). Average precipitation was 1,008 mm, ranging from 806 mm (2011) to 1,277 mm (2014). Irrigation was provided in spring to control frost and during summer (with the exception of 2014, when summer irrigation was not needed) by overhead sprinklers. This additional water, approx. 200 mm/year on average, prevented any possible water deficit stress events. As a result, SWC was often close to the maximum soil water‐holding capacity.

During the 6‐year study period, the observed H_2_O and CO_2_ fluxes displayed a clear seasonal pattern with a maximum in summer. In spring, NEE fluxes started to increase (become more negative) later than ET and displayed a more even distribution of maximal sink values around −4.9 g C day^−1^. Interestingly, the wet 2014 year showed the highest maximal daily sink (−6.0 g C day^−1^) and the lowest maximal ET (2.6 mm/day), therefore representing the maximum water use efficiency in the observational period.

#### 3.1.2 Observed average patterns of measured and calculated variables at daily and annual timescales

**Figure 2:**
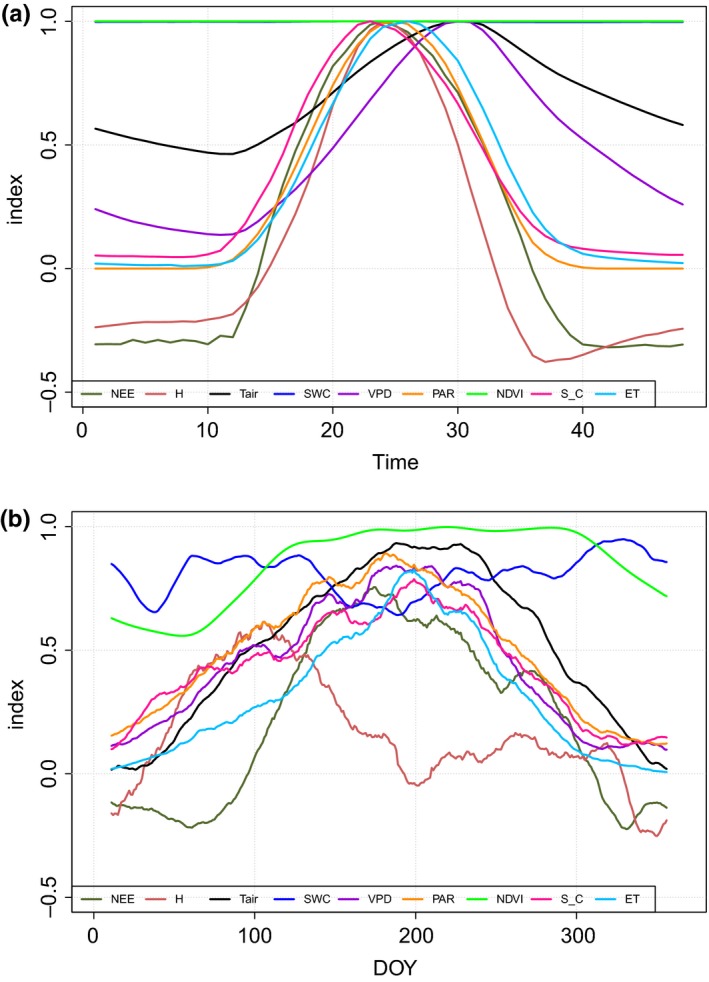
Normalized average patterns, with moving average, of the environmental drivers (PAR; VPD; Tair; SWC), other stand variables (canopy conductance, sensible heat, NDVI) and observed biological fluxes (ET; NEE) at daily (a) and annual scales (b). Data, shown in the time domain, represent averages of the 6 years

Analyzing the average pattern at the scales of earth rotation (day, Figure 2a) and revolution (year, Figure 2b), we can understand how the different physical and biological variables are related to the sun–earth interaction. In Figure 2, all the considered variables are normalized to ease the pattern recognition. During the day, the first variable to reach its maximum is the stand canopy conductance (11:30 hr). Then, in the range of 1 hr around midday, PAR, NEE, and ET reach their maximum when maximal stand canopy conductance is already decreasing. Temperature and VPD maximum occur at 15:00 hr. NEE is perfectly lined with the maximum radiation, which occur in the interval between 12:00 and 12:30 local standard time. SWC does not show any notable variability at this scale.

At the annual scale (Figure 2b), NEE and PAR are not tightly correlated as they are at the daily scale. In spring, NEE starts to increase later than ET and grows more rapidly as compared to PAR. PAR and NEE on average reach their maximum in the same period, in the first half of July (DOY 192, Figure 2b). The maximum in ET occurs a few days later, in mid‐July (DOY 196), 2 weeks after the maximum in VPD (DOY 182), while the air temperature average maximum is observed in late August (DOY 233). At the annual scale, the most asymmetric pattern is displayed by sensible heat (H) which reaches its maximum on DOY 108 (April), when the vegetation cover, as indicated by the low NDVI, is still far from its maximum.

The stand canopy conductance, the VPD and the ET show a similar annual pattern, with a broad maximum in the central part of the year. *T* lags all other variables (maximum at DOY 233, August 20). SWC displays an irregular pattern with its minimum in July (DOY 185) and maximum in November (DOY 320).

### 3.2 Correlation between the observed variables

#### 3.2.1 Hysteresis between observed variables at daily and annual timescales

**Figure 3:**
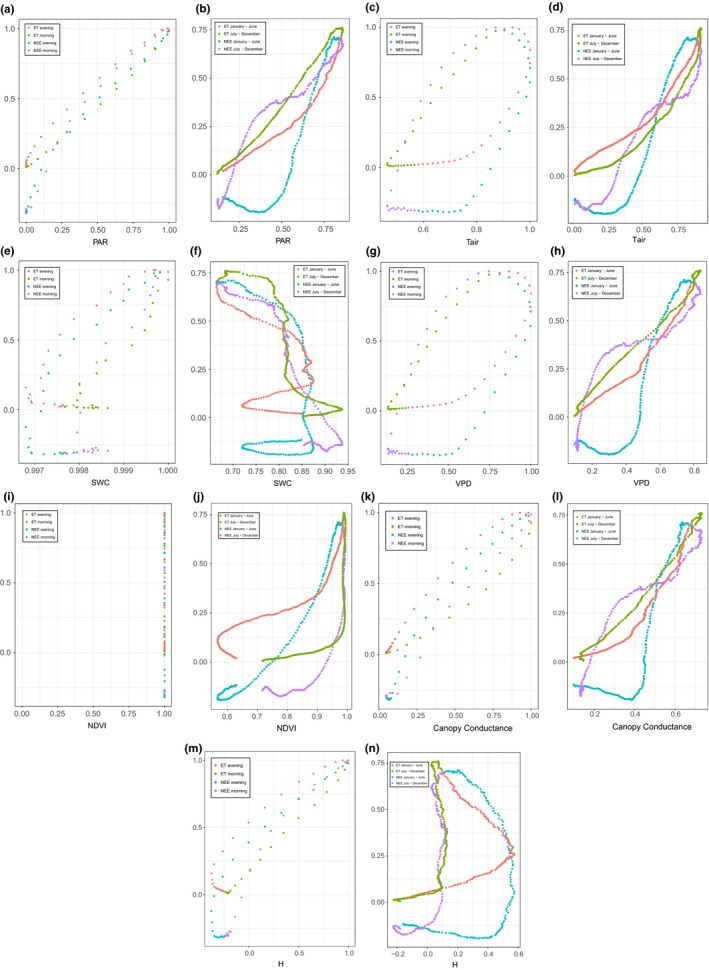
Normalized relationships at daily and annual scale between main environmental drivers and observed fluxes. Left side panels (a, c, e, g, i, k, m) show the correlations at daily scale, right side panels (b, d, f, h, j, l, n) show the correlations at annual scale. Same data and variables as in Figure 2, but shown in the environmental variable domain. First and second half of the datasets are shown in different colors

Using the same dataset composed by daily and annual averaged patterns as used in Figure 2, we can evaluate the relationship between observed variables and the considered fluxes in the variable's domain. In Figure 3, with a few exceptions, we observe positive relations between environmental parameters and biological fluxes (ET, NEE) with variable counterclockwise, rate‐dependent hysteresis (see Zhang et al., 2014). At a given intensity of the parameter value, we generally observe larger flux values in the morning at the daily scale and in spring at annual scale. In some cases, the hysteresis is wide: Notably, an ample rate‐dependent hysteresis is observed at daily scale between *T*, VPD and ET, NEE (Figure 3c,g) while PAR and VPD show a wide hysteresis with NEE (and not with ET) at annual scale (Figure 3b,h). Even wider is the hysteresis between H and both ET and NEE at annual scale (Figure 3n).

Exceptions are the relations of SWC with both ET and NEE, which is partly time dependent and negative at daily scale and irregular at annual scale. The relation between PAR and NEE shows an almost bijective correspondence at daily scale (Figure 3a), while clockwise hysteresis is found only between canopy conductance and both ET and NEE at daily scale (Figure 3k), and between NDVI and both ET and NEE at annual scale (Figure 3j).

#### 3.2.2 Wavelet coherence analysis

By means of the wavelet decomposition, we can analyze the coherence between the NEE and ET and the other environmental variables and identify the common oscillatory behavior of fluxes and drivers at the different scales, from half‐hour to year. With the analysis of the phase, we can further indicate if the oscillations of drivers precede or follow those of biological fluxes.

**Figure 4:**
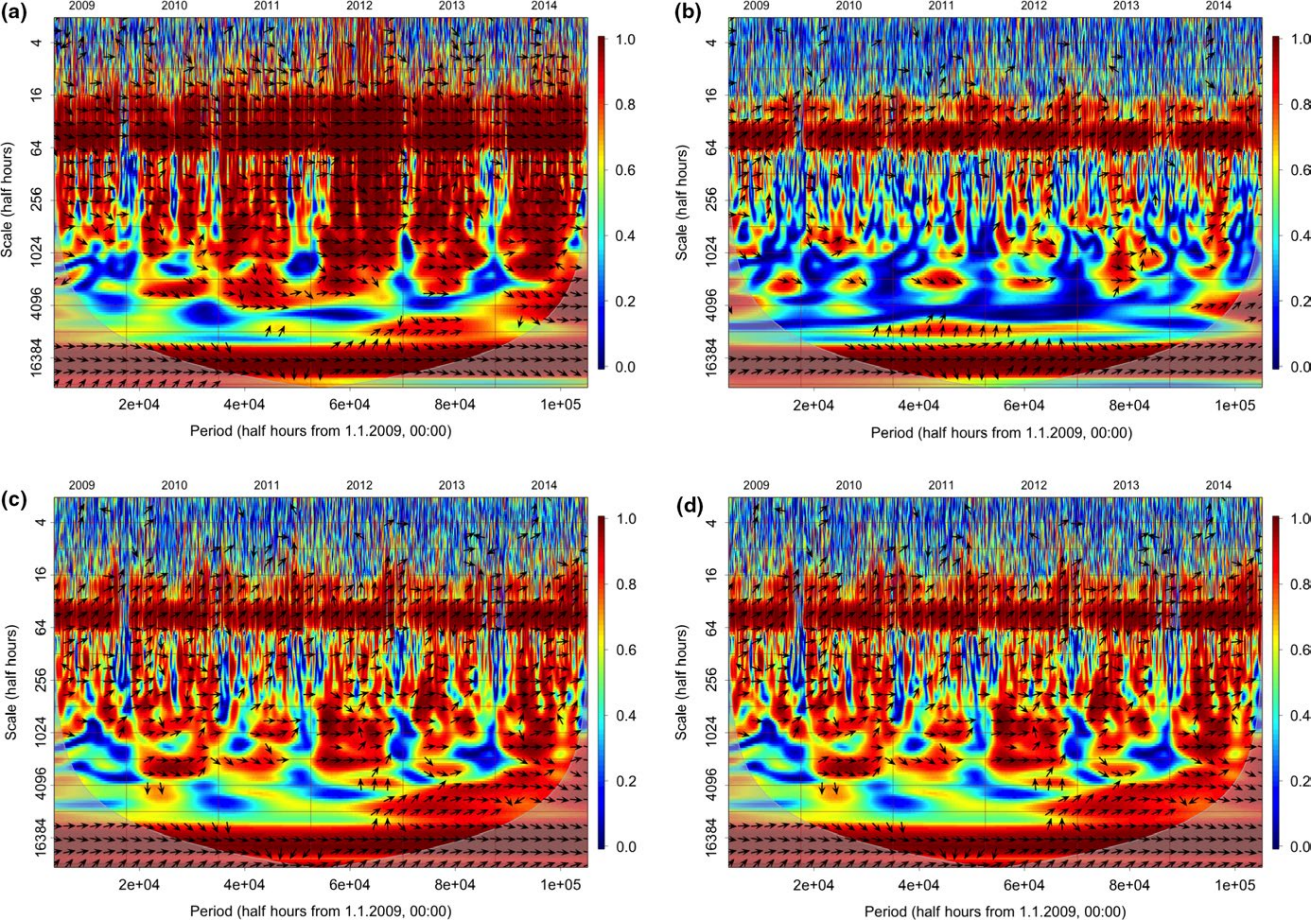
Wavelets coherence analysis between observed ET fluxes and environmental drivers: (a) PAR, (b) *T*, (c) SWC, (d) VPD. *X* axis represents the time period of observation (6 years), the *y* axis represents the frequency of the waves, from multiannual at the bottom to half hourly at the top. Fading colors in the bottom outside a round part indicate the not‐significant part of the image. Red color indicates the highest correlation, blue represents the minimum and yellow intermediate values; arrows indicate the presence of statistically significant correlation. Arrows angle (trigonometric convention) indicates the phase relationship: horizontal arrows indicate that the variables are in phase, inclined arrows indicate that a phase shift exists between the variables

The wavelet coherence analysis between PAR and ET shows a very strong correlation at daily and subdaily scales (more than 16 half hours, Figure 4a); the variables are tightly in phase at these scales. Increasing the scale, the correlation becomes generally weaker and does not apply to the entire season. Only at annual scale (see bottom of the figure), the correlation between the two variables shows again high significance.

The correlation between *T* and ET (Figure 4b) is weak at subdaily scale, but it increases at scales higher than the day; a shift in phase indicates that the ET phase precedes that of *T*. At the annual scale, the correlation becomes significant and in phase. In Figure 4c, we can observe the relationship between ET and SWC. As expected, these variables are not significantly correlated at any scales, and we cannot identify any phase. In Figure 4d, we can observe the correlation between ET and VPD. At daily and subdaily scales, the correlation and the phase are quite similar to those of *T*, indicating a relatively strong coherence with a shift in phase, as also VPD, like *T*, follows ET peaks. We can observe interesting periods of significant coherence between these two variables at scales from daily to seasonal in all the years except 2011. Similarly to *T*, the coherence becomes strong and in phase at the annual scale.

**Figure 5:**
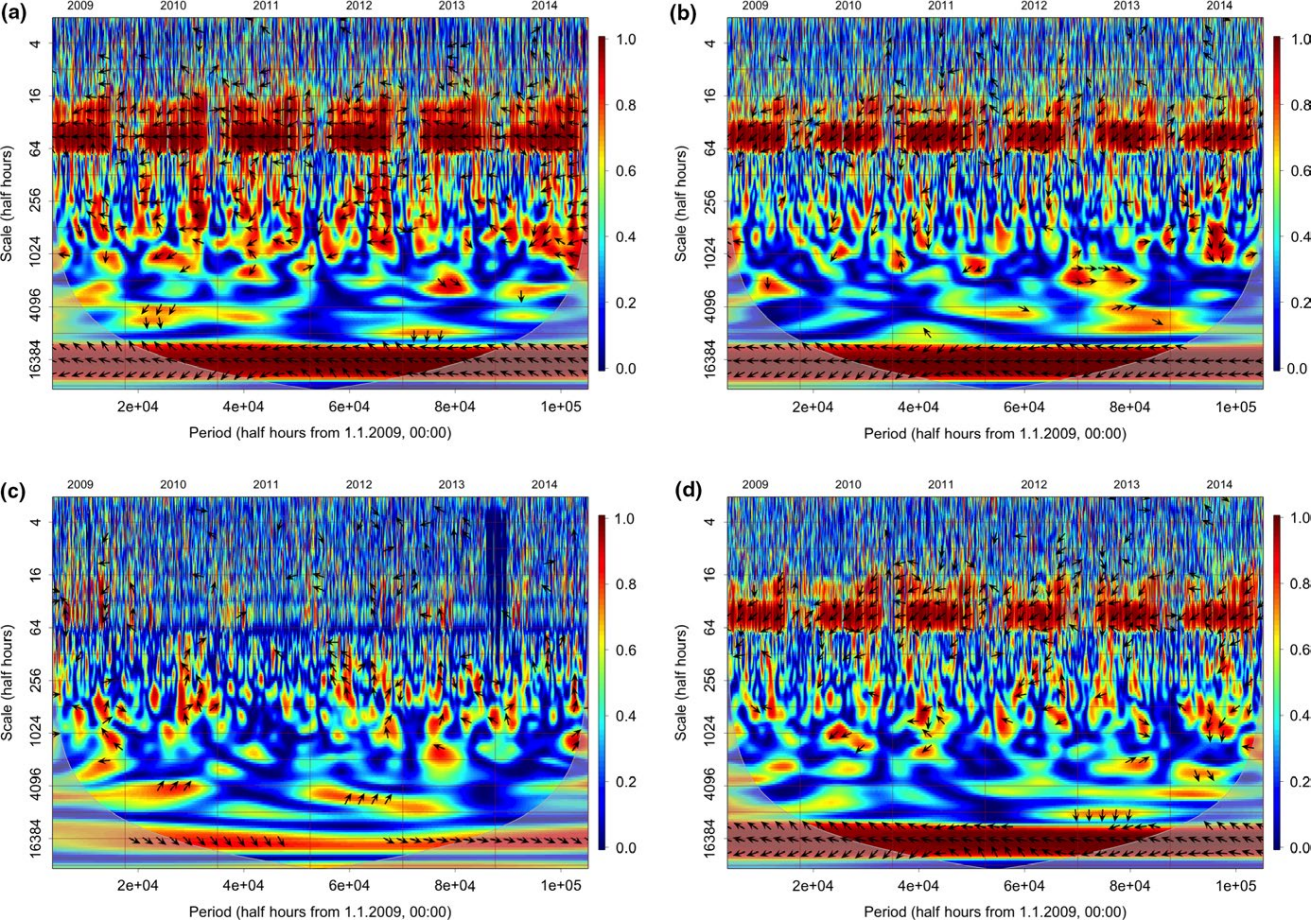
Wavelets coherence analysis between observed NEE fluxes and environmental drivers: (a) PAR, (b) *T*, (c) SWC, (d) VPD. Same conventions as for Figure 4

In Figure 5, we can observe the correlation between NEE and the same environmental drivers. Overall, the coherence between NEE with environmental drivers is similar to that shown by ET, but somehow weaker. Given the presence of both positive (respiration) and negative (assimilation) NEE values, the phase can be either positive (autumn and winter) or negative (summer). The PAR and NEE (Figure 5a) are tightly in antiphase at the daily scale only, and nearly completely in antiphase at the annual scale. The coherence between NEE and *T* (Figure 5b) is similar to that found for ET, with strong coherence not only at the daily scale but also at the annual scale. Also in this case, the variables are not in phase, NEE precedes *T* and shows opposite correlation in spring–summer with respect to autumn–winter periods. The correlation with SWC (Figure 5c), as expected, is always very weak, and the phases are undistinguishable. In the case of VPD (Figure 5d), the correlation is weaker with NEE than for ET, but still quite strong. The variables are not in phase at the daily scale: NEE precedes VPD at the daily scale, but becomes in phase and negative, therefore indicating a positive influence on assimilation at the annual scale.

#### 3.2.3 Relationship between environmental constraints and observed ET and NEE at different timescales

Through the analyses at different timescales of the relationships between environmental drivers and biological fluxes (ET, NEE), it is possible to evaluate how these relations evolve, and possibly change, in the different time‐periods used to group the data.

**Figure 6:**
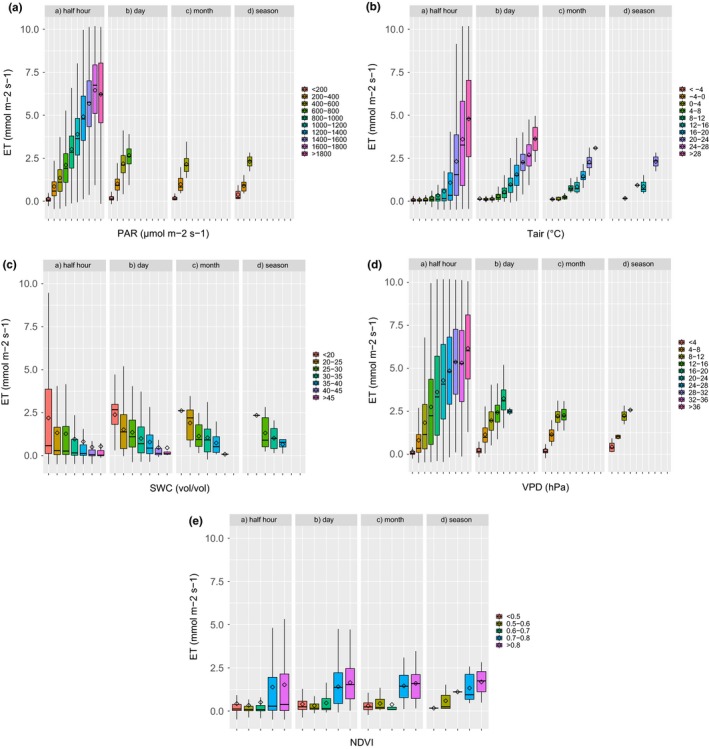
Relations between environmental drivers (PAR,* T*, SWC, VPD) and satellite NDVI versus observed ET fluxes. Boxplots represent the 50% distribution percentile, vertical bars represent 99% distribution. Same colors represent same distribution interval in the different timescales. (a) PAR, (b) *T*, (c) SWC, (d) VPD, (e) NDVI

The relationships between environmental variables and ET are graphically displayed in Figure 6 and evaluated numerically in Table 1. We can observe that the relationship between PAR and ET is positive and monotonic along the entire time ranges. The two‐parameters equation used to model their relationship (Equation (2)) is significant at half‐hourly (*r* ^2^ = 0.82) and daily scales (*r* ^2^ = 0.76), but not at larger scales. PAR represents the highest correlated parameter with ET at half hour and daily scales, and only the third at monthly and seasonal scales. Conversely, the three‐parameters equation describing the relationship between *T* and ET (Equation (3)) increases its strength increasing the timescale, with the *r* ^2^ increasing from 0.52 (hh) to 0.89 (season). This relationship is clearly logistic at short timescales, with a high level of scattering, while at larger scales it becomes almost linear and the scattering decreases. The linear relationship of SWC with ET (Equation (6)) is quite surprisingly negative at all the timescales, but the *r* ^2^ is, however, low. The relationship between VPD and ET is generally positive along the entire range, with a saturation effect when the VPD approaches its maximum; at half‐hour scale, these two variables are correlated with *r* ^2^ of 0.54; the *r* ^2^ increases at the larger scales, reaching values of 0.88 at seasonal scale. The relationship between NDVI and ET do have a biological interpretation at monthly and seasonal scales only, as the satellite data were collected beweekly. At these scales, the correlation is low, but increasing with scale (*r* ^2^ = 0.34 at seasonal scale).

**Table 1:** Linear or nonlinear modeling of the relationships between environmental variables (VPD; PAR; *T*, SWC) and NDVI and the observed evapotranspiration (ET) fluxes at different timescales (half hour, day, month, and season). Equations are reported with ET suffix in the text

ET versus environmental variables													
Relation	Equation	Timescale											
		Half hour			Day			Month			Season		
		Coefficients	*r* ^2^	*p*	Coefficients	*r* ^2^	*p*	Coefficients	*r* ^2^	*p*	Coefficients	*r* ^2^	*p*
ET versus PAR	*f* = *ax*(1 − EXP(−*xb*))	*a* = 0.0036; *b* = 0.2078	0.819	*a* < 0.0001; *b* = 0.0002	*a* = 0.0047; *b* = 0.0046	0.757	*a* < 0.0001; *b* < 0.0001	*a* = 0.0040; *b* = 1.0000	0.754	*a* = 1.0000; *b* = 1.0000	*a* = 0.0041; *b* = 1.000	0.8	*a* = 1.0000; *b* = 1.0000
ET versus *T*	*f* = *a*/(1 + (*b*)EXP(*cax*))	*a* = 7.2640; *b* = −0.0336; *c* = 455.5747	0.524	*a* < 0.0001; *b* < 0.0001; *c* < 0.0001	*a* = 3.8038; *b* = −0.0483; *c* = 39.4934	0.696	*a* < 0.0001; *b* < 0.0001; *c* < 0.0001	*a* = 3.9729; *b* = −0.0447; *c* = 37.2315	0.824	*a* = 0.0070; *b* = 0.0914; *c* = 0.0025	*a* = 4.4493; *b* = −0.0368; *c* = 31.8336	0.89	*a* = 0.3219; *b* = 0.5164;*c* = 0.0190
ET versus SWC	*f *= *ax* + *b*	*a* = −0.078; *b* = 3.3855	0.08	*a* < 0.0001; *b* < 0.0001	*a* = −0.0647; *b* = 3.1357	0.13	*a* < 0.0001; *b* < 0.0001	*a* = −0.0847; *b* = 3.7630	0.20	*a* = 0.0002; *b* < 0.0001	*a* = −0.0973; *b* = 4.1443	0.24	*a* = 0.0059; *b* = 0.0326
ET versus VPD	*f *= *ax*(1 + 1/(*b*EXP(*xc*)))	*a* = 0.2047; *b* = −0.5602; *c* = 0.2275	0.54	*a* < 0.0001; *b* < 0.0001; *c* < 0.0001	*a* = 0.1847; *b* = 42.8775; *c* = 95.5848	0.727	*a* < 0.0001; *b* = 1.0000; *c* = 1.0000	*a* = 0.1915; *b* = 116.165; *c* = 44.3644	0.80	*a* = 1.0000; *b* = 1.0000; *c* = 1.0000	*a* = 0.199; *b* = 312,794; *c* = 15,243.5	0.88	*a* = 1.0000; *b* = 1.0000; *c* = 1.0000
ET versus NDVI	*f* = *a*_ET_ *x *+ *b*_ET_	*a* = 3.8413; *b* = −1.6514	0.072	*a* < 0.0001; *b* < 0.0001	*a* = 4.0408; *b* = −1.7271	0.249	*a* < 0.0001; *b* < 0.0001	*a* = 4.1793; *b* = −1.8233	0.328	*a*=<0.0001; *b* = 0.0014	*a* = 4.2962; *b* = −1.8854	0.34	*a* = 0.0087; *b* = 0.0829

**Figure 7:**
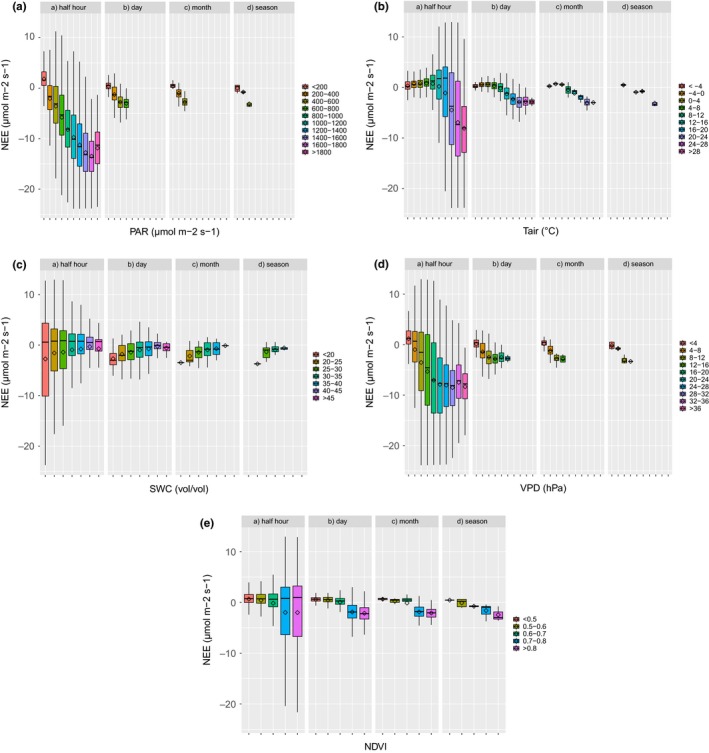
Relations between environmental drivers (PAR,* T*, SWC, VPD) and satellite NDVI versus observed NEE fluxes, with the same graphical conventions as in Figure 6. (a) PAR, (b) *T*, (c) SWC, (d) VPD, (e) NDVI

The relationships between environmental variables and –NEE at different timescales are shown in Figure 7 and Table 2. At half‐hour scale, the relationship between PAR and –NEE shows the well‐known nonrectangular hyperbolic relationship, with the saturation effect, however, just about visible. At higher timescales, the parameter indicating the maximum assimilation capacity becomes not significant and the saturation effect less evident. Although the *r* ^2^ generally increases with timescales, similarly to what is observed for ET, the significance of the regression between PAR and –NEE decreases. At half‐hour timescale, the relationship of air *T* with –NEE shows values around zero at temperature below 4°C, and an ample scatter along a logistic response curve at higher temperature values. At this scale, the *r* ^2^ is very low (0.19). Increasing the scale, the relationship between *T* and –NEE becomes nearly linear, and the *r* ^2^ increases. At seasonal scale, the *r* ^2^ is 0.89, and *T* is the most correlated variable. The relationship between SWC and –NEE is barely visible, with the lowest values (negative, assimilation) in the lowest range, indicating an apparent negative role of SWC in the carbon sequestration capacity at all the scales. The relationship between VPD and –NEE is almost logistic at half‐hourly timescale, with an increasing but saturating function, with 90% of –NEE attained at 20 hPa, a large scattering and a low *r* ^2^ (0.26). This relationship becomes more linear and the correlation coefficient increases at seasonal scale, making VPD the second best‐correlated parameter with –NEE, according to Equation (9) (*r* ^2^ = 0.72). The relationship between NDVI and –NEE increases with scales and reaches a value of *r* ^2^ = 0.34 at seasonal scale.

**Table 2:** Linear or nonlinear modeling of the relationships between environmental variables (VPD; PAR; *T*, SWC) and NDVI and the observed NEE fluxes at different timescales (half hour, day, month, and season). Equations are reported with net ecosystem exchange (NEE) suffix in the text

–NEE versus environmental variables													
Relation	Equation	Timescale											
		Half hour			Day			Month			Season		
		Coefficients	*r* ^2^	*p*	Coefficients	*r* ^2^	*p*	Coefficients	*r* ^2^	*p*	Coefficients	*r* ^2^	*p*
NEE‐ versus PAR	*f* = (*axb*)/(*ax *+ *b*) + *c*	*a* = 0.0166;*b* = 60.8269;*c* = −2.3294	0.5846	*a* < 0.0001; *b* < 0.0001; *c* < 0.0001	*a* = 0.0073; *b* = −5,787; *c* = −1.0213	0.495	*a* < 0.0001; *b* = 1.0000; *c* < 0.0001	*a* = 0.0075; *b* = −50.9412; *c* = −1.2217	0.673	*a* = 0.0207; *b* = 0.8352; *c* = 0.0051	*a* = 0.0041;*b* = −31,126;*c* = −1.1878	0.7301	*a* = 0.2449; *b* = 1.0000; *c* = 0.1975
NEE‐ versus *T*	*f* = *a*/(1 + (*b*)EXP(*cax*)) + *d*	*a* = 4.5323;*b* = −0.0210;*c* = 4.3223;*d* = −2.5021	0.1938	*a* = 1.0000; *b* = 1.0000; *c* = 1.0000; *d* < 0.0001	*a* = 3.8038; *b* = −0.0483; *c* = −0.2046; *d* = −7.6391	0.474	*a* = 1.0000; *b* = 1.0000; *c* = 1.0000; *d* < 0.4430	*a* = 4.5844; *b* = −0.0074; *c* = 0.2901; *d* = −4.5200	0.764	*a* = 1.0000; *b* = 1.0000; *c* = 1.0000; *d* < 0.6921	*a* = 4.1296;*b* = −0.0157;*c* = 2.4144;*d* = −1.8639	0.8984	*a* = 0.0063; *b* = 0.9979; *c* < 0.0001; *d* = 0.3838
NEE‐ versus SWC	*f* = *ax* + *b*	*a* = −0.1088;*b* = 4.6638	0.0100	*a* < 0.0001; *b* < 0.0001	*a* = −0.0863; *b* = 3.8467	0.067	*a* < 0.0001; *b* < 0.0001	*a* = −0.0863; *b* = 3.8467	0.067	*a* < 0.0001; *b* < 0.0001	*a* = −0.1433;*b* = 5.6209	0.2025	*a* = 0.0532; *b* = 0.0192
NEE‐ versus VPD	*f* = *ax*/(*b*EXP(*bx*)) + *c*	*a* = 0.0115;*b* = 0.0146;*c* = −2.2792	0.2621	*a* < 0.0001; *b* < 0.0001; *c* < 0.0001	*a* = 0.0305; *b* = 0.0528; *c* = −1.0373	0.393	*a* < 0.0001; *b* < 0.0001; *c* < 0.0001	*a* = 0.0115; *b* = 0.0251; *c* = −1.1233	0.62	*a* = 0.4692; *b* = 0.3317; *c* = 0.0072	*a* = 0.0004;*b* = 0.0013;*c* = −0.8541	0.7467	*a* = 0.9806; *b* = 0.9803 *c* = 0.2263
NEE‐ versus NDVI	*f* = *ax* + *b*	*a* = 9.9206;*b* = −5.4690	0.0308	*a* < 0.0001; *b* < 0.0001	*a* = 8.7599; *b* = −4.9937	0.339	*a* < 0.0001; *b* < 0.0001	*a* = 8.8294; *b* = −5.0389	0.483	*a* < 0.0001; *b* < 0.0001	*a* = 8.1190;*b* = −4.5088	0.4693	*a* = 0.0087; *b* = 0.0829

#### 3.2.4 Modeling the relationships among the observed variables at the different timescales

The multiple regression analysis revealed that both NEE and ET could be predicted by different numbers and types of environmental parameters depending on the temporal scales at which the data are arranged (Table 3). The *r* ^2^ values increase always moving from shorter (half hour) to longer (seasonal) temporal scale. Half‐hour data of both NEE and ET are affected by several parameters, but most of the variability was accounted for by PAR, followed by VPD and air *T*. At the daily scale, NEE variability could be explained mainly by four predictors, air *T*, PAR, VPD, and NDVI, while ET by three of them: *T*, PAR, and VPD. Soil moisture, although significant, always explains a relatively small fraction of the total variability of the response factors at daily scale. Both at monthly and seasonal scales, the only significant predictor of NEE data is air *T*. Monthly ET data are predicted by both VPD and air *T*, while VPD is the only significant predictor of ET data at the seasonal scale (Table 3).

**Table 3:** Relaimpo analysis indicating the minimum adequate model variables, and their parameters, at the different timescales, for evapotranspiration (ET) and net ecosystem exchange (NEE)

Flux (*y*)	Timescale	*n*	*r* ^2^	Minimum adequate model^a^	Relative importance of the parameters (sum = 1)
ET (mmol H_2_O m^−2 ^s^−1^)	Half hour	92,640	0.78	*y* = −0.671 + 0.003*x *+ 0.074*w* − 0.005*k* + 0.863*j*	*x* = 0.62; *z* = 0.01; *w* = 0.33; *k* = 0.01; *j* = 0.03
	Day	1,930	0.8	*y* = −0.277 + 0.002*x* + 0.028*z* + 0.060*w* − 0.008*k* + 0.292*j*	*x* = 0.33; *z* = 0.25; *w* = 0.30; *k* = 0.04; *j* = 0.08
	Month	63	0.84	*y* = −0.295 + 0.042*z* + 0.141*w*	*z* = 0.47; *w* = 0.53
	Season	19	0.9	*y* = −0.246 + 0.230*w*	*w* = 1.00
NEE (μmol CO_2_ m^−2^ s^−1^)	Half hour	92,640	0.56	*y* = 4.785 − 0.011*x* + 0.067*z* − 0.008*w* − 0.016*k* − 4.448*j*	*x* = 0.74; *z* = 0.08; *w* = 0.15; *k* = 0.01; *j* = 0.02
	Day	1,930	0.6	*y* = 2.656 − 0.007*x* − 0.050*z* + 0.148*w* + 0.023*k* − 3.671*j*	*x* = 0.33; *z* = 0.25; *w* = 0.18; *k* = 0.03; *j* = 0.21
	Month	63	0.75	*y* = 1.196 − 0.182*z*	*z* = 1.00
	Season	19	0.85	*y* = 1.138 − 0.180*z*	*z* = 1.00

a *x* = PAR (μmol m^−2^ s^−1^); *z* = Tair (°C); *w* = VPD (hPa); *k* = SWC (m^3^/m^3^); *j* = NDVI (–).

## 4 DISCUSSION

### 4.1 Analysis of the multiannual dataset composed by half‐hourly data

The availability of databases encompassing timescales ranging from minutes to years, as recently made available by the spread in the use of the EC technique, has allowed the study of the temporal effects on the observed relationships between biological fluxes and environmental variables. In this study, we have analyzed several environmental variables that, with the exception of soil water availability, significantly varied at both small‐ and large‐temporal scales.

Using linear and nonlinear regressions, wavelet coherence and phase shift analyses, we answered two main questions: (1) whether the relation between weather variables and biological fluxes changes with the time of integration and (2) whether the modeling capability of the meteorological variables showing a hysteretic relation with biological fluxes increase with timescale. The answers highlight the specific functional properties of the considered agricultural ecosystem, and have a relevant modeling consequence.

#### 4.1.1 Does the relation between environmental drivers and biological fluxes change across timescales?

Our results clearly indicate that timescale strongly affects the way environmental parameters are related to either NEE or ET. To explain this finding, we have to consider that the multiple environmental variables are differently aggregated according to the scale. Sun–earth interactions are the main driver of the relationships between environmental factors and biological functions. The effects of environmental parameters on the exchange of carbon dioxide and water vapor are mediated by the heating and the cooling of atmosphere, vegetation and soil, and by the time needed to develop the biological structures (e.g., LAI) and biochemical compounds (e.g., chlorophyll content), which occur separately at different scales. Any relationship is inherently entangled with those produced by other drivers. The apparent relationship at a given scale is therefore influenced by the different combination of concurrent variables.

#### 4.1.2 Can we improve the modeling capability of fluxes showing a hysteretic response to environmental drivers by increasing the time of integration?

We tested the hypothesis that increasing the time of integration increases the overall modeling capability, as hysteretic and delayed responses to environmental drivers are increasingly captured. We made this hypothesis considering the stabilizing effect of the management on the agricultural systems (Lauri et al., 2009; Zanotelli et al., 2015). Therefore, we supposed an opposite pattern with respect to what was found in natural ecosystems, where the direct effect of environmental drivers loses importance while increasing the timescale, in favor of biologically mediated interactions (Richardson et al., 2007; Wu et al., 2015).

Besides a preliminary technical reason, that is, the reduction in random errors influencing half‐hourly data more than aggregated ones (Goulden, Munger, Fan, Daub, & Wofsy, 1996), we can postulate two main reasons to explain these differences: (1) the capture of progressively larger parts of delayed responses and (2) the reduction of hysteretic effects at the longer timescales.

On the one hand, by extending the time window beyond a given interaction, it is possible to capture a progressively larger part of the delayed responses of any single variable. Increasing the scale, we can progressively capture the delayed effects of stomatal openness, the maturation of the photosynthetic machinery, and the enhanced photosynthates demand during fruit maturation.

On the other hand, in the analysis of the interactions between drivers and fluxes, we can observe that interactions are generally not in phase in the time domain. These out‐of‐phase interactions correspond to “rate‐dependent” hysteresis in the variable domain (Zhang et al., 2014). In some cases, the environmental forcing occurs before the peak in the ecosystem flux and induced delayed response. This is the case, for instance, of the spring rise in temperature, that precedes the ET, and more markedly, the NEE rise. In other cases, the peak of the environmental forcing takes places after the flux peak. In fact, both ET and photosynthesis directly respond to radiation, while other environmental drivers such as temperature, although having a generally positive influence on fluxes, frequently lag by a time larger than that of the flux response.

### 4.2 Correlations between drivers and fluxes at the different scales

The effects of the combined interactions, in phase and not in phase, can be simply evaluated through the variation of the correlation coefficients between fluxes and drivers in different integration periods.

At half‐hourly scale, both linear (used in the Relaimpo analysis) and nonlinear regressions (used in custom‐made nonlinear functions) indicate PAR as the most important driver of ET and NEE, almost completely in phase. At this scale, the correlation of PAR with both ET and NEE was strong, and the phase shift limited. Instead, the relationship between VPD and ET showed a clear hysteresis in the variable domain. This hysteresis was explained mechanistically by Bohrer et al. (2005) and by Matheny et al. (2014) as an effect of hydraulic stress occurring in the afternoon.

At the daily scale, most of the effects related to daily phase shift disappear, but delayed effects are yet minimally taken into account. At this scale, as it can be seen by linear and nonlinear analyses (Tables 1 and 2), the PAR begins to lose part of its relevance in the flux determination, in favor of drivers that are not in phase at subdaily scales, such as temperature and VPD. This is also the scale where the wavelet analysis shows the highest correlation for most of the drivers.

At the monthly scale, part of the delayed effects linked to leaf development (LAI; chlorophyll content; effect of fruit load on photosynthesis) is taken into account. Although the wavelet analysis does not show any specific correlation at this scale, the correlation coefficients in linear and nonlinear regressions generally increase. This is particularly evident for *T* and NEE and for both *T* and VPD and ET (Tables 1 and 2), while the relative importance of PAR consistently decreases (Table 3).

At the seasonal scale, the effects of leaf development are mostly taken into account. At this scale, the radiation increasingly loses its relevance and its modeling capability in favor of drivers that, at daily and seasonal scale, show not‐in‐phase effects, like temperature and, notably, VPD. Linear and nonlinear regressions show some difference in the output, but the increased relevance of the drivers occurring not in phase with fluxes at half‐hourly scale, like *T* and VPD, is always evident (Matheny et al., 2014; Wohlfahrt & Galvagno, 2017; Zhang et al., 2014).

### 4.3 Consequences for modeling

Ecosystem functioning is the result of multiple processes having various speed and response time. Interpreting and reconstructing these interactions across time is a main challenge for ecosystem modeling (Wu et al., 2015); regardless of the fact that mechanistic or empirical approaches are used, a correct representation of the correlations between environmental drivers and fluxes at the different scales is always needed. The mechanistic method is thought to be more suitable in this regard (Rastetter, Aber, Peters, & Ojima, 2009) because each underlying process is represented at the proper timescale. However, in our study, we obtained increasing modeling performance applying an empirical method, introducing temporal aggregation of drivers and responses.

In order to take into account both delayed and anticipated correlations two possible computational strategies can be used in empirical models. First, one can model separately these effects: The well‐established approach of thermal sum, or other phenological modeling approaches based on spring warming (Chuine, Cour, & Rousseau, 1999; Jolly, Nemani, & Running, 2005; Melaas et al., 2013) are examples that show how a delayed effect of temperature on vegetation can be considered. The second approach is to capture delayed effects, or anticipated ones, together with current interactions by extending the time window of data integration. In this way, out‐of‐phase effects are increasingly captured as the timescale increases.

In our study, we showed that a simple average at a given scale can improve the correlations between drivers and processes at the same scale. A similar result was found by Razavi, Elshorbagy, Wheater, and Sauchyn (2015), who used a moving average to better correlate driver and output.

Studies in temperate and tropical ecosystems (Richardson et al., 2007; Wu et al., 2015) showed that an opposite pattern can be observed, and long‐term hysteresis due to biological responses can take place, reducing the correlation with meteorological drivers at the longer scales. Also in managed ecosystems, care should be taken to avoid overaveraging: in fact, statistical significance and part of ecosystem signaling are lost when increasing the averaging time. Long‐term data records are necessary to obtain averaged data with statistical significance at annual or multiannual scale. This observation highlights the relevance of long‐term monitoring infrastructures, such as LTER, NEON, or ICOS, that provide the potential for exploring these relationships at larger scales (i.e., annual to decadal) in the different ecosystem types (Chu, Baldocchi, John, Wolf, & Reichstein, 2017).

We suggest that current difficulties in modeling some ecosystem phenomena, such as the lagged effect of drought (Liang et al., 2015; Von Randow et al., 2013), the linkage between photosynthesis and decomposition process (Stoy et al., 2009), or the ecosystem resilience (Johnstone et al., 2016) depend on the large hysteretic and lagged effects that can be captured only along annual or multiannual integration periods.

In this study, we have quantitatively demonstrated the importance of different drivers on carbon and water cycling at multiple timescales. Interactions among variables at short scales do have little forecast capabilities with respect to conveniently aggregated long‐term data records. The dataset used, although quite large, is still not able to be used to infer in a statistically significant way the relationships between the meteorological drivers and the biological fluxes at scales larger than seasonal. The findings as obtained through the Relaimpo analysis show that with increasing timescale, the number of variables needed to represent the observed biological fluxes decrease, and largely change when the timescale increases. This has relevant and largely unexplored consequences for scale dependent ecological modeling. The observation that radiation decreases its influence when increasing the considered timescale suggests a decreasing ability of the commonly used light use efficiency models to describe the variability in the fluxes at the longer scales.

The power of satellite products such as NDVI to describe the seasonal variability in the fluxes appears to be limited, and mostly confined to the identification of the green‐up during spring. The capacity of the VPD to predict the fluxes, and particular ET, at the longer scales reflects the key role of this driver in regulating the gas exchange between the considered vegetation and the atmosphere. This suggests that VPD can be a good predictor of ET from medium to longer timescales.

## 5 SUMMARY AND CONCLUSIVE REMARKS

This study presents the results from the longest data series of meteorological, satellite, and EC‐based biological fluxes measured above an irrigated apple orchard. The dataset is unique in providing consistent observations of an economically relevant woody crop over 6 years.

The analysis encompassing the average daily and annual patterns of constraint on the fluxes, the wavelet coherence analysis and the nonlinear regression modeling, clearly shows that not‐in‐phase interactions between environmental drivers exist, in particular between *T* and VPD and observed biological fluxes. Extending the time of the integration window, the effect of the phase shift in the time domain, corresponding to a hysteresis in the environmental variables domain, tend to vanish. Similarly, the correlations tend to be more linear and less scattered when extending the integration time.

At the smaller scales (half hour, day), several variables have to be taken into account to model the observed fluxes, and the modeling capability is relatively low. Instead, at the larger scales (month, season), we were able to model up to 90% of the observed variability in the fluxes with a reduced number of variables (one or two). In particular, while PAR is the most effective parameter to model half‐hour and daily interactions, the VPD is particularly effective in describing the observed monthly to seasonal variability in ET, and *T* is particularly effective in explaining NEE at the same scales.

This study showed a marked difference in the relevance of explanatory variables at the different scales. While in natural ecosystems the biological variability tends to be dominating at timescales larger than 1 month (Richardson et al., 2007; Wu et al., 2015), we observed an increasing explanatory capacity of meteorological constraints also at the largest scales (seasonal), and only a small increase in correlation with biological‐related variables (NDVI). We hypothesize that this feature is tied with agronomic practices tending to maximize the crop performances and stabilize its relations with the environment.

This work suggests that, when biological‐related variables are not relevant, a statistical elaboration of input data to reduce the impact of phase shift and hysteresis among variables and fluxes can improve the modeling capacity of some explanatory variables, particularly those, like *T* and VPD, which are not in phase with observed fluxes. Finally, the observation about the changing variables needed to explain the observed fluxes in natural and managed ecosystems at the different timescales opens new questions and perspectives regarding environmental modeling in a changing climate.

## CONFLICT OF INTEREST

The authors declare no conflict of interest.

## AUTHORS' CONTRIBUTION

ET, LM, and MT conceived the study. DZ, ET, and LM performed the analysis. LM wrote the first draft of the text. All the authors contributed to the final version of the manuscript.
